# Influence of the Microbiome on Chronic Rhinosinusitis With and Without Polyps: An Evolving Discussion

**DOI:** 10.3389/falgy.2021.737086

**Published:** 2021-10-01

**Authors:** Kyle S. Huntley, Joshua Raber, Lauren Fine, Jonathan A. Bernstein

**Affiliations:** ^1^Dr. Kiran C. Patel College of Allopathic Medicine, Nova Southeastern University, Fort Lauderdale, FL, United States; ^2^Department of Internal Medicine, Division of Immunology/Allergy Section, University of Cincinnati College of Medicine, Cincinnati, OH, United States

**Keywords:** chronic rhinosinusitis, nasal polyp, allergic rhinitis, non-allergic rhinitis, microbiota

## Abstract

Chronic rhinosinusitis (CRS) is widely prevalent within the population and often leads to decreased quality of life, among other related health complications. CRS has classically been stratified by the presence of nasal polyps (CRSwNP) or the absence nasal polyps (CRSsNP). Management of these conditions remains a challenge as investigators continue to uncover potential etiologies and therapeutic targets. Recently, attention has been given to the sinunasal microbiota as both an inciting and protective influence of CRS development. The healthy sinunasal microbiologic environment is largely composed of bacteria, with the most frequent strains including *Staphylococcus aureus, Streptococcus epidermidis*, and *Corynebacterium* genera. Disruptions in this milieu, particularly increases in *S*. *aureus* concentration, have been hypothesized to perpetuate both Th1 and Th2 inflammatory changes within the nasal mucosa, leading to CRS exacerbation and potential polyp formation. Other contributors to the sinunasal microbiota include fungi, viruses, and bacteriophages which may directly contribute to underlying inflammation or impact bacterial prevalence. Modifiable risk factors, such as smoking, have also been linked to microbiota alterations. Research interest in CRS continues to expand, and thus the goal of this review is to provide clinicians and investigators alike with a current discussion on the microbiologic influence on CRS development, particularly with respect to the expression of various phenotypes. Although this subject is rapidly evolving, a greater understanding of these potential factors may lead to novel research and targeted therapies for this often difficult to treat condition.

## Introduction

The human microbiota plays a crucial role in the preservation of human health, and investigations into this complex ecosystem have become a key focus in medical research (1). Defined as a community of microorganisms within a specific environment, the microbiota generally serves a mutualistic purpose in the human body; organisms gain a nourishing milieu while the human host receives benefits such as improved immunologic development and metabolic function (2–4). However, when disturbed, this previously mutualistic symbiosis can transform into a destructive process, with consequences affecting multiple organ systems. A prominent example of this phenomenon is the gut-lung axis, whereby disturbances of the intestinal microbiota are implicated in pathologic changes of the respiratory system (5, 6). Specifically, decreases in gut bacterial diversity have been hypothesized to shift innate immunologic responses from a Th1 to a Th2 predominance, leading to atopic disease potentiation, including asthma and allergic rhinitis (7, 8). Discovery of these microbiologic connections have spurred investigations into previously unexplored microbiologic milieu, including the upper airway, and their contributions toward disease (5).

Upper airway diseases that are now hypothesized to be linked to microbiologic processes include rhinitis and chronic rhinosinusitis (CRS) (9). Rhinitis represents a heterogenous syndrome which may be acute or chronic, and is characterized by at least one of the following symptoms: nasal congestion, rhinorrhea (anterior and posterior), sneezing, and nasal itching (10). Rhinitis can additionally be classified into allergic (AR) or non-allergic (NAR) subtypes which are differentiated by clinical history and the presence of an IgE-mediated inflammatory reaction in response to allergen exposure. In contrast, CRS is defined as a clinical syndrome classified by at least 12 weeks of inflammation of the sinunasal mucosa which results in facial pain and/or pressure, hyposmia/anosmia, nasal drainage, and nasal obstruction (11, 12). This latter syndrome is classified into two subtypes, CRS with nasal polyps (CRSwNP) and CRS without nasal polyps (CRSsNP), each with diverse pathologic mechanisms (13). CRS is widely prevalent within the US and Europe and thus has a significant socioeconomic and quality of life impact (14). Although there is some overlap between the clinical manifestations of pure rhinitis (particularly AR) and CRS, each are driven by differing pathologic mechanisms and potential influences of the local microbiota. Definitive treatment and understanding of CRS remain elusive, but recent technological advances in microbiologic characterization have highlighted both the protective and pathogenetic roles of the nasopharyngeal and sinus microbiotas. A greater microbiologic understanding of CRS can guide clinicians toward more focused treatment and encourage future therapeutic research efforts.

Here, we provide a broad overview of the current pathophysiologic understanding of both CRSwNP and CRSsNP and how microbiologic influences in resident bacteria, fungi, and viruses, may contribute to disease formation. Because this subject matter is rapidly evolving, the purpose of this review is to provide a thorough discussion on the nasopharyngeal and sinus microbiotas regarding risk factors, current treatments, and future therapeutic directions.

### Pathogenesis of CRS With and Without Nasal Polyps

**Figure 1:**
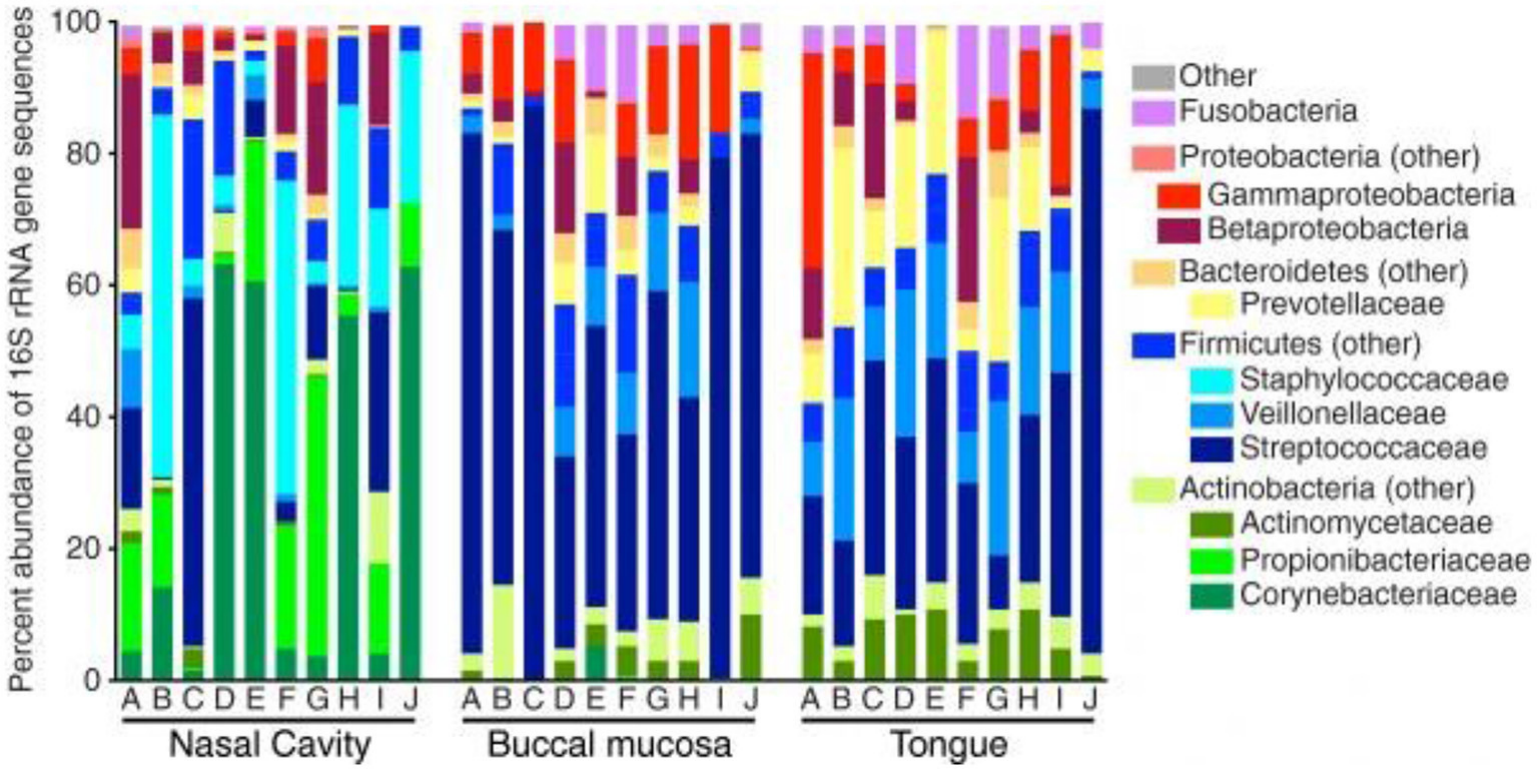
Bacterial composition within the healthy nasal cavity, compared to the buccal mucosa and tongue. Data presented as relative abundances, and each column (marked by letters A through J) represents an independent study participant. Reprinted from (22), with permission under the Creative Commons Attribution License.

Various theories regarding the underlying pathogenesis of CRS exist, including underlying host responses to airborne fungal elements, superantigenic exotoxins produced by *Staphylococcus*, pro-inflammatory biofilms, interruption of the sinunasal microbiome, and defects in the host barrier with inappropriate immune responses (Figure 1) (13, 15). Which of these theories is correct, or whether individual host factors interplay between one or more of these, is still unclear. However, the immune barrier hypothesis is the most inclusive for all of the components of all of these hypotheses as it involves impaired host defense, colonization by bacteria and fungi, loss of barrier function, an increased local innate and adaptive immune response, as well as local autoimmune response (15). Nasal polyps are a potential sequalae of this inflammatory reaction and occur in up to 30% of patients with CRS (16). Originally investigators believed this reaction was driven by type 2 inflammation, but recently this theory has been re-evaluated as the potential role of type 1 mediators in nasal polyps continues to expand (16). Treatment of each CRS subtype is typically aimed at the downstream inflammatory response, regardless of the underlying initiating pathophysiology (12).

### Classifying the Healthy Nasal Cavity Microbiota

#### Bacterial Environment

The mucus membranes within the body provide an ideal environment for microbial nourishment (17). The characterization of bacteria within these environments can be accomplished with either culture-dependent or culture-independent analyses. Culture-dependent analyses rely on direct growth and identification of bacteria from a desired sample. Culture-dependent techniques are successful in identifying the most prominent bacterial colonizers, but many bacterial species are unable to grow outside of their natural body microhabitat and represents a limitation to this detection method (18, 19). Recently, advancements in culture-independent genetic sequencing and molecular analyses have resulted in the precise microbiologic characterization of the nasopharynx and sinus cavities. Typically, culture-independent techniques identify and amplify the 16s rRNA gene within a desired sample (19). The 16s rRNA gene is conserved within the bacterial genome, but each taxa demonstrates genetic variability within this region; thus, specific taxa can be identified from available genetic databases (20). Additionally, the 16s rRNA genome can be used to identify similar operational taxonomic units (OTUs) which are commonly used to quantify bacterial diversity (19).

The Human Microbiome Project (HMP), funded by the National Institutes of Health, was a foundational investigation which characterized the healthy bacterial microbiota and verified the presence of opportunistic pathogens within the anterior nares (21). Samples were collected from the anterior nares of 242 healthy participants which then underwent 16S rRNA gene profiling analysis. The opportunistic bacterium *Staphylococcus aureus* and *Staphylococcus epidermidis* were present in 29 and 93% of samples, respectively. Other notable opportunistic genera such as *Propionibacterium, Corynebacterium*, and *Moraxella* were highly abundant in select healthy anterior nares samples, although their prevalence varied. In contrast, the often-virulent pathogen *Pseudomonas aeruginosa* was absent in all anterior nare samples.

Subsequently, more recent studies have further examined the microbiota deeper within the nasal cavity. Bassis et al. evaluated the middle meatus in healthy adults via culture-independent 16S rRNA gene analysis with 269 sequences per sample (22). Comparable to the anterior nares from the HMP, *Corynebacteria* were highly abundant within the middle meatus, constituting at least half of the bacterial profile in 40% of the participants and were ubiquitous in the remaining subjects (Figure 1). *Propionibacterium* were also ubiquitous, ranging from 0.4 to 42.4% abundance. *Staphylococcaceae* genera were also present in all subjects, ranging from 2.2 to 55.0% abundance, although differentiation between the species *aureus* and *epidermidis* could not be determined. Notably, *Moraxellacae* genera were not widely prevalent, and were absent in 40% of participants. These genera have been noted to be more prevalent in children compared to adults (23). Other larger culture-independent analyses have found comparable distributions in the middle meatus of healthy adults, with a high prevalence of *Staphylococcaceae, Propionibacterium*, and *Corynebacterium* as well as the detection of the opportunistic pathogens *Streptococcus pneumoniae, Moraxella catarrhalis*, and *Haemophilus influenzae* in select individuals (24).

#### Emerging Fungal and Viral Nasal Microbiotas

Fungi and viruses are noted components of the healthy human flora, but knowledge regarding their contributions toward the nasal microbiota is evolving (25–27). Within the anterior nares, the fungal species *Malassezia* predominates (28). Similarly, *Malassezia* is highly prevalent within the sinonasal cavity, with an average abundance of 53.9% (29). *Cladosporium* (5.96%) and *Pleosporles* (2.17%) were the next most abundant fungal species, with the remaining community composed of a range of diverse species (29). Notably, *Malassezia restrica* abundance has an inverse relationship with *Propionibacterium*, which may imply a competing relationship. (29).

Symptomatic viral infections of the upper airway have been shown to alter the homeostatic bacterial community, but investigations into the viral microbiota (e.g., the virome), of asymptomatic and healthy individuals continues to be explored (30). Asymptomatic hosts may possess common viruses such as rhinovirus, enterovirus, and annellovirus within the respiratory tract microbiome (31). Bacteriophages have also been isolated within the anterior nares, including those that infect *Propionibacterium* (32). Phages of the human virome have demonstrated an immunomodulatory effect and can influence the composition of the bacterial microbiota (17). Overall, the literature on the healthy nasal virome and fungal contributions is limited and remains an important topic for future investigations. Table 1 compares the common bacterial, viral and fungal microbiota in healthy normal individuals to subjects with allergic rhinitis, CRSwNP and CRSsNP.

**Table 1:** Comparison Of Common Bacteria, Viruses And Fungi In Normal, Allergic Rhinitis, CRSwNP And CRSsNP.

	**Bacteria**	**Fungi**	**Viruses**
Healthy	Propionibacterium spp. (21, 22, 24, 25) Corynebacterium spp. (21, 22, 24, 25) Moraxella spp. (21, 23) Staphylococcus spp. (21, 22, 24, 25) Streptococcus pneumoniae Moraxella catarrhalis Haemophilus influenza	Malassezia (29) Cladosporium (30) Pleosporles (30)	Rhinovirus (32) Enterovirus (32) Annelovirus (32)
CRSwNP	Corynebacterium spp. (13, 33) Staphylococcus spp. (13, 33) Streptococcus spp. (13, 33) Citrobactere spp. (13) Haemophilus influenzae (33) Enterobacter spp (33)		
CRSsNP	Corynebacterium spp. (13) Staphylococcus spp. (13) Streptococcus spp. (13) Haemophilus influenzae (34)		
Allergic Rhinitis	Spirochaete spp. (35) Pseudomonas spp. (35) Peptostreptococcaceae spp. (35)		

## Comparing the Microbiotas of CRSwNP and CRSsNP

The pathogenetic mechanisms of CRSwNP and CRSsNP are diverse, and thus it has been hypothesized that differing microbiotas may influence disease formation. Systematic reviews have further demonstrated that the nasal microbiota differs significantly in diseased states (19). Recently, Wei et al. compared the bacterial composition of the middle meatus in CRSwNP patients, CRSsNP patients, and patients without a diagnosis of CRS (13). In this culture-dependent investigation, the most commonly isolated strains from 136 cases of CRSwNP were *Corynebacterium* (19.9%), *S. epidermidis* (19.1%), *Streptococcus* (14.7%), and *S. aureus* (11.0%); the most prominently isolated strains in the 66 CRSsNP cases were *Corynebacterium* (21.2%), *S. epidermidis* (21.2%), *S. aureus* (13.6%), and *Streptococcus* (7.6%). Similarly, these four gram-positive bacteria composed the majority of isolated strains in the 17 control cases, and when compared across the three study cohorts, there was no statistically significant difference in abundance. The only strain identified as statistically distinct across the three study groups was *Citrobacter*, with a 5.9% isolation rate found in CRSwNP and no isolated strains found in the CRSsNP and control groups (*p* = 0.034). However, when the CRSwNP group was further stratified by eosinophil percentage in the peripheral blood, *S. aureus* was more common in the elevated eosinophil subgroup compared to the normal eosinophil percentage subgroup (17.2 vs. 3.3%; *p* = 0.011). These findings are agreeable with previous studies which concluded an association between *S. aureus* in the middle meatus and CRS with allergic asthma (36). In contrast, Wei et al. found that *S. epidermidis* decreased as eosinophil percentages increased (13). Asthma was also associated with increased isolation rates of *Corynebacterium* and *Pseudomonas* within the CRSwNP population. There were no associated differences in bacteria isolation rates within the CRSsNP and control populations when controlled for eosinophilia or asthma. Fungal isolates were relatively uncommon and insignificant across cohorts (13).

The results from Wei et al. are comparable to other published reports investigating the nasal microbiota across the spectrum of CRS (34, 37). For example, Niederfuhr et al. concluded statistically insignificant differences in abundance of the most common bacterium, coagulase-negative *Staphylococcus, Corynebacterium*, and *S. aureus*, across CRS study groups (37). Although not statistically significant, the authors found *S. aureus* more commonly in CRSwNP compared to CRSsNP (23 v. 8%) and *H. influenzae* was more commonly detected in CRSsNP (31 v. 13%) compared to CRSwNP (37). The authors from Niederfuhr et al. did not further stratify the study groups to investigate potential associations with eosinophilia, asthma, or other related characteristics. In contrast, Liu et al. stratified their CRSwNP cohort by peripheral eosinophil percentage and concluded an inverse association between eosinophilia and isolates of gram-negative aerobic and facultative anaerobic bacteria (34).

These microbiologic analyses provide insight into the potential associations between microbiota and CRS development. Although the most common bacteria were proportionally similar across CRS cohorts in these culture-dependent investigations, stratification by blood eosinophil level and asthma identified potential associations with CRSwNP and certain pathogens, specifically *S. aureus* and *S. epidermidis*. This stratification permits us to view CRS as a more continual spectrum of disease, rather than a binary process, and spurs hypotheses regarding the role of specific microbiota on immune modulation and pathogenesis. Furthermore, multivariate correspondence analyses demonstrate a potential positive association between CRSwNP and *S. aureus, Streptococcus, Haemophilus, Enterobacter*, and *Corynebacterium*, which was not apparent on observational chi square analyses (34). Similarly, culture-independent genetic analyses assert a greater abundance of *S. aureus* within CRSwNP patients compared to CRSsNP, which has influenced recent hypotheses explaining CRSwNP pathogenesis (33).

### Microbiota of Allergic Rhinitis

Whereas CRS is generally associated with decreased microbiologic diversity, the available literature demonstrates AR may instead be associated with *increased* bacterial diversity relative to NAR and CRS cohorts (8, 38). In a study by Choi et al., the authors collected samples from the middle meatus and vestibule of 19 seasonal AR patients and 20 NAR patients. Outside of the allergy season, each cohort demonstrated comparable numbers of unique bacterial phyla (*p* < 0.14); however, during the symptomatic allergy period, the AR cohort reported significantly increased unique bacterial phyla compared to the NAR cohort (*p* < 0.036), although the authors did not report which specific bacterium differed. Further, the authors demonstrated a positive correlation between unique bacterial phyla in the middle meatus and nasal eosinophil count within the AR cohort during the allergy season (ρ = 0.35 and *p* < 0.033), suggesting increased Th2 inflammation with bacterial diversity. Lal et al. compared bacterial species diversity within the middle meatus across CRSwNP, CRSsNP, and AR patients; their findings demonstrated that while AR and CRSwNP patients exhibited similar levels of bacterial diversity, each group was associated with higher number of unique bacterial phyla compared to CRSsNP (*p* < 0.05) (39). Gan et al. further identified increased *Spirochaete* abundance within AR nasal cavities compared to CRS patients, while the AR cohort also exhibited higher abundances of *Pseudomonas* and *Peptostreptococcaceae* than in control cohorts (*p* < 0.005) (40). However, in contrast to Choi et al., the authors did not collect AR samples during times of increased allergen exposure, which may represent a limitation to making direct comparisons between studies.

### Role of the Nasopharyngeal Microbiome in CRS and Polyp Development

#### Bacteriologic Influences

**Figure 2:**
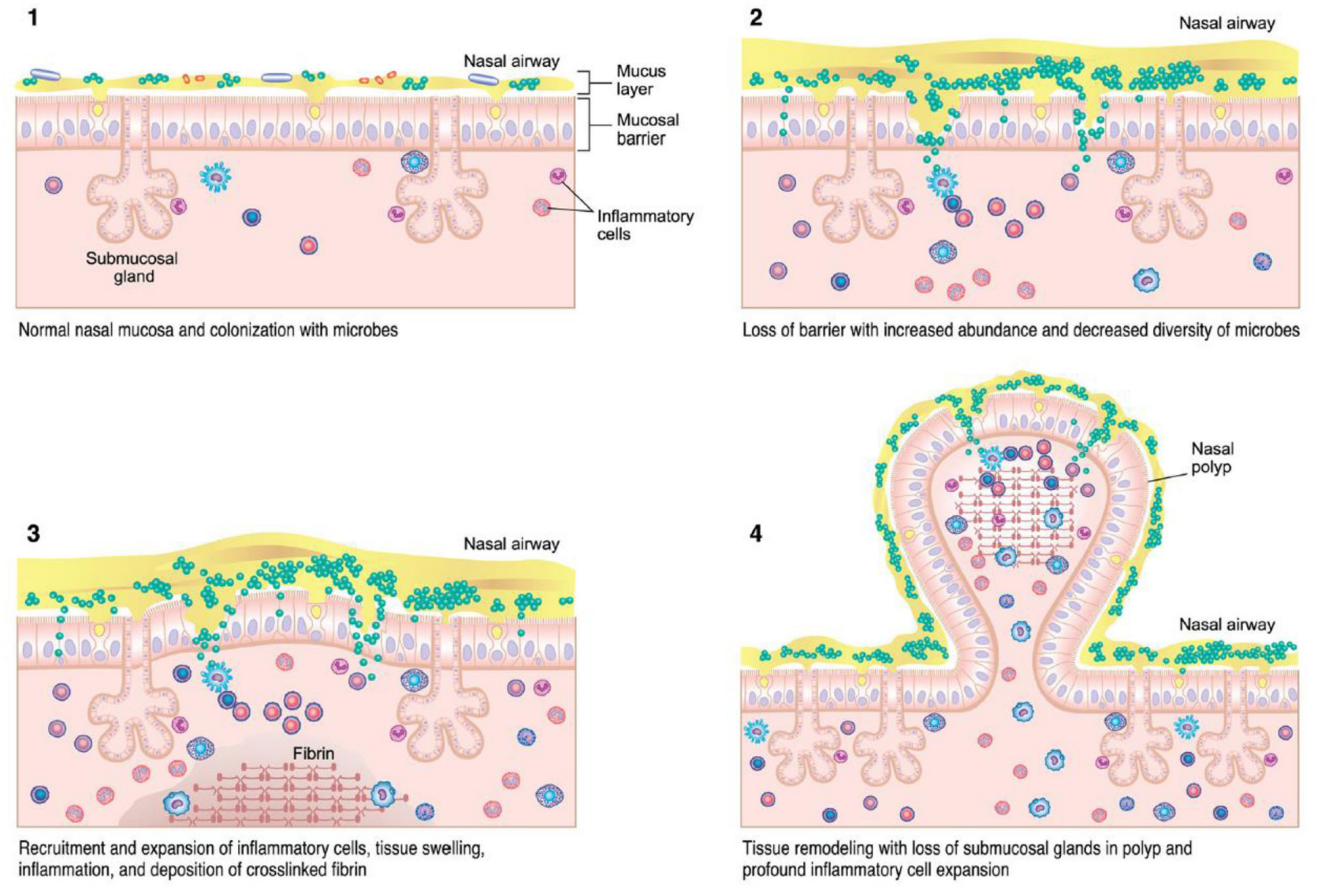
CRSwNP immunopathogenesis (13) – colonization of microbes with accumulation of immune cells can lead to tissue injury, inflammation, and mucosal barrier loss in CRS.

CRS, by its definition, is driven by chronic inflammation at the mucosal surface. The nasal microbiota may contribute toward the pathogenesis of CRS and influence the inflammatory mechanism between Th1 or Th2 predominance (Figure 2). *S. aureus* has been one of the most extensively studied promoters of this underlying reaction (35, 36). Multiple hypotheses have attempted to explain this relationship, including a superantigen-induced local T-lymphocyte reaction with resultant IgE production (41). In this proposed mechanism, *S. aureus* releases superantigens which induces a potent local T-cell reaction. This cascade leads to production of Th2 cytokines such as IL-4, IL-5, and IL-13. The subsequent eosinophilia and B-cell activation results in specific IgE production against the *S. aureus* superantigens, further perpetuating the chronic Th2 inflammatory reaction (42, 43). Asthma, a common comorbidity in CRS patients, has also been associated with increased formation of specific IgE directed against the *S. aureus* superantigen (44).

In contrast, other experimental models have associated *S. aureus* with Th1 and Th17 inflammation, which is classically described in CRSsNP, further complicating the definitive role this bacterium may play in CRS development (35, 45). In this mechanism, *S. aureus* releases extracellular membrane vesicles (EV) which contain receptors, lipids, and nucleic acids, among other bioactive compounds (35, 46). These EVs induce subsequent Th1 and Th17 cytokine responses, leading to a predominately neutrophilic inflammatory reaction; in addition, *S. aureus* EVs do not cause augmentation of IL-4, a potent Th2-cytokine. Other *S. aureus* virulence factors such as surface protein A and lipoteichoic acid (LTA) have similarly been shown to induce histamine release and promote epithelial colonization, respectively (42, 47). Therefore, it is likely *S. aureus* plays a pathologic role in both CRS phenotypes and thus requires further investigation into whether it is functioning as a potential facilitator for both Th1 and Th2 chronic inflammation. These observations also promote the hypothesis that the CRS T-lymphocyte cascade occurs along a spectrum, rather than a strict binary Th1 or Th2 fashion as classically described (48, 49).

Alterations in nasal bacterial diversity have also been reported, which may also contribute toward CRS pathogenesis (50). Culture-based approaches, such as those discussed previously in the context of CRS bacterial abundance, are adequate for isolating prominent species but lack in their ability to estimate total microbiologic diversity (9, 51). Thus, genetic sequencing has been utilized to quantitatively assess these differences. Using 16s rRNA sampling, Abreu et al. concluded similar bacterial burdens within the maxillary sinuses of CRS patients and healthy controls (2.10 × 10^6^ vs. 2.92 × 10^6^ rRNA gene copies per microgram; *p* = 0.37), but found a significant decrease in species diversity within the CRS cohort (~1,200 vs. ~950 unique bacterial taxa in healthy and CRS patients, respectively; *p* < 0.008) (50). It is necessary to note some participants in this investigation had a history of antibiotic use, and although the authors minimize the role of this potential confounder on the results, previous studies have implicated antibiotic therapy with decreased bacterial diversity and an increased *S. aureus* abundance (36). In a study conducted by Chalermwatanachai et al. where antibiotic use was an exclusion criteria, the authors nevertheless concluded a significant decrease in bacterial diversity in CRSwNP patients compared to healthy controls; CRSsNP patients were not included in this analysis (52). In metagenomic investigations directly comparing bacterial diversity between CRSwNP and CRSsNP patients, the nasal cavity of the CRSsNP cohort demonstrated significantly decreased bacterial species diversity, and each CRS phenotype (wNP and sNP) possessed significantly fewer unique bacterial species compared to healthy controls (33).

The precise influence of species diversity on CRS development remains under investigation. Proposed mechanisms include a loss of “protective” bacteria and the formation of biofilms. One bacteria hypothesized to serve a protective role is *Propionibacterium acnes* (52). Laboratory models demonstrate *P. acnes* releases propionic acid, which attenuates macrophage-induced inflammation and decreases growth of *S. aureus* (53, 54). One genetic investigation concluded *P. acnes* is more common in healthy individuals compared to CRSwNP, but the detected levels of propionic acid were not high enough to exert these protective effects *in vivo* (52). Nevertheless, *P. acnes* remains a bacterium of interest as well as others including *Lactobacillus sakei, Dolosigranulum* genera, and *Citrobacter* genera. (13, 50, 55) *Lactobacillus* species are capable of producing lactic acid which may outcompete pathogenic species (50). *Dolosigranulum* genera have been associated with decreased rates of respiratory infections and abundance is inversely related to *S. aureus* colonization (55). *Citrobacter* is a common intestinal bacterium that is also noted to be significantly more abundant within the nasal cavity of CRSwNP patients compared to CRSsNP, spurring potential investigations into potential immune-protective properties (13). Bacterial dysbiosis, such as reductions in bacterial diversity with concomitant abundances of *S. aureus* and *Pseudomonas*, are also hypothesized to influence the dynamics of nasal biofilms (56, 57). These biofilms confer a high resistance to antimicrobial defenses and can result in long-standing inflammation at the mucosal surface (58). Although present in both healthy and CRS patients, their presence had been associated with poorer treatment outcomes (59).

#### Viral Influences

Respiratory viruses such as rhinoviruses, coronaviruses, and influenza viruses are routinely found within the nasal cavity of CRS patients, but relatively few studies have examined these viral associations between CRS phenotypes (60, 61). In one study conducted by Goggin et al., the authors demonstrated the aforementioned respiratory viruses are associated more with CRSsNP populations, compared to CRSwNP and healthy controls (62). However, the authors concluded no difference in disease severity between CRS phenotypes. Multiple proposed mechanisms for viral influence on CRS development exist and further investigations are warranted. Current evidence implies that replicating respiratory viruses may damage epithelial barriers, which facilitates environmental exposure and perpetuates inflammation (63, 64). Other proposed mechanisms include an impairment of ciliary function and mucus overproduction (65, 66). It remains unclear if viral influences perpetuate chronic inflammation within CRS or contribute to the initial disease stimulus (67).

### Modifying Disease Risk

Current research supports that a reduction in the proportion of potentially protective strains of bacteria may decrease the stability of the sinunasal microbiome and ultimately contribute to disease progression, severity, and CRS phenotype (68, 69). Therefore, modifying certain risk factors and targeted treatments may shape tailored therapy in patients with CRS (70). For example, literature has supported the use of probiotic applications to decrease the density of certain bacteria, including pathogenic strains of *Staphylococcus aureus*, associated in high concentrations with the microbiome of CRS (71–73). These effects have also been seen with the use of intranasal probiotic irrigation (74). Furthermore, evidence suggests that certain probiotic strains have the potential to have immunomodulatory effects that favor the stimulation of a Th1 profile, further augmenting humoral immunity with increases in IgA production and enhanced mucosal defense immune responses (75). Another potential method to modify disease risk by further altering the nasal microbiome is the use of intranasal corticosteroids; the use of intranasal corticosteroids has been shown to modify the commensal microbiota and may further serve to decrease the density of pathogenic strains of colonizing bacteria related to the development of CRS (76).

CRS pathogenesis also involves the increased presence of local inflammatory mediators due to mucosal irritation (77). The risk for CRS may be reduced by increasing the consumption of anti-inflammatory foods and decreasing the frequency of meat and fat intake (78). Tobacco smoke, a local irritant to the nasal mucosa, is thought to be involved in the pathogenesis and symptom burden of CRS (79). For example, patient smoking history has been determined to have a substantial influence on sinunasal bacterial colonization and modification of nasal mucosal physiological and immunological properties (80).

## Limitations

As previously mentioned, our understanding of the microbiologic influences on CRS is rapidly expanding but still incompletely understood (1). Thus, it is challenging for a single review to comprehensively capture all recent developments and perspectives. Additionally, as demonstrated by multiple systemic analyses, there is notable variation in the microbiologic landscape between research studies (19, 81). Such variation is present across both healthy and CRS subjects, indicating lack of generalizability of these studies as the microbiome is likely affected by a spectrum of environmental and host determinants; factors such as these may complicate participant selection in large-scale investigations (82, 83). In addition, a lack of conformity in methods for collection of specimens, culture techniques and in DNA/RNA extraction can contribute to the variability of results reported (84, 85). Other factors such as antibiotic use, disease severity, and patient age must also be controlled for when investigating the microbiome (86).

## Conclusions

CRS is a clinical syndrome which has a broad scope of clinical presentations and treatment response. It is clear that CRSwNP and CRSsNP represent two distinct phenotypes and that CRSwNP is likely driven by Type 2 inflammation; yet questions still remain on how the nasal cavity microbiome influences these divergent clinical conditions.

Treatment of CRS is currently aimed at reducing inflammation and obstructive defects by either medical or surgical modalities. Current medical therapy for both CRS phenotypes may include nasal saline irrigation and intranasal corticosteroids, while operative therapies such as functional endoscopic sinus surgery may help restore sinus ventilation (87–89). However, recent approval by regulatory agencies for biologic therapies that target Type 2 inflammation including dupilumab (an anti-IL-4α monoclonal antibody) (90), omalizumab (an anti-IgE monoclonal antibody), and mepolizumab (anti-IL-5) have been found to be efficacious at reducing nasal polyps in patients with CRSwNP. This has resulted in controversial treatment paradigm shifts regarding when to use these agents. Should they be used in lieu of surgery or be reserved for use post-operatively in order to achieve the maximum desired effect? (91) Further well-controlled studies will be needed to answer this question that will also need to factor in economic costs of surgery and biologic use.

nProbiotics may play an important treatment role in CRS via modification of the GI-respiratory axis resulting in attenuation of underlying inflammation (75, 92) as they can alter the host-microbiome composition and immune function, including the production of antimicrobial peptides and compounds (72). In addition, alteration of the host-microbiome composition can result in a microbial-driven shift in development of immunomodulatory cells and cytokines such as Foxp3+ T reg cells and IL-10, respectively (72). However, studies that have used probiotics to modify clinical symptoms in CRS and other airways diseases have been mixed, with most unable to produce a detectable long-term improvement in clinical symptoms (93, 94).

Rhinitis may present similarly to CRS, and investigations comparing their respective microbiologic environments are ongoing. The current literature implies AR may be associated with increased bacterial species diversity compared to NAR and CRS (38). Like CRS, AR treatments are aimed at attenuating the underlying Type 2 inflammation using intranasal corticosteroids and antihistamines (95). As discussed, biologics are effective in patients with CRSwNP and other comorbid allergic conditions such as asthma but less is known how they might affect the nasal microbiome in AR or other chronic rhinitis subtypes (96). Allergen immunotherapy (AIT) is another effective treatment modality for AR (97). However, studies investigating changes to the underlying microbiota during AIT have been unable to correlate symptomatic improvement with detectable changes in nasal microbial diversity (38). Interestingly, the nasal microbiota following symptomatic improvement with AIT does not resemble the microbiota of a non-atopic healthy individual (38).

In summary, this review is not intended to be an exhaustive assessment of all research pertaining to CRS and the microbiome, but rather to illustrate the current understanding and gaps in knowledge pertaining to this important area of research. As with many chronic inflammatory diseases, the underlying pathophysiology of CRS and chronic rhinitis is a complex interplay between genetics, barrier function, immune function and the resident microbiome composition and balance. Targeted approaches that can favorably alter the pathogenic microbiome will likely become an important part of a multifaceted treatment approach for CRS.

## Author Contributions

KH, JR, and LF contributed toward the writing of the manuscript. KH led the design and implementation of the manuscript outline. KH, JR, LF, and JB provided research and literature necessary for the review. JB provided final supervision and ensured the accuracy of all texts.

## Conflict of Interest

The authors declare that the research was conducted in the absence of any commercial or financial relationships that could be construed as a potential conflict of interest.

## Publisher's Note

All claims expressed in this article are solely those of the authors and do not necessarily represent those of their affiliated organizations, or those of the publisher, the editors and the reviewers. Any product that may be evaluated in this article, or claim that may be made by its manufacturer, is not guaranteed or endorsed by the publisher.
